# Influence of Solvents' Polarity on the Physicochemical Properties and Photocatalytic Activity of Titania Synthesized Using *Deinbollia pinnata* Leaves

**DOI:** 10.3389/fchem.2020.597980

**Published:** 2020-12-03

**Authors:** Yakubu Rufai, Sheela Chandren, Norazah Basar

**Affiliations:** ^1^Department of Chemistry, Faculty of Science, Universiti Teknologi Malaysia, Johor Bahru, Malaysia; ^2^Department of Chemistry, Federal College of Education (FCE), Okene, Nigeria; ^3^Centre for Sustainable Nanomaterials, Ibnu Sina Institute for Scientific and Industrial Research, Universiti Teknologi Malaysia, Johor Bahru, Malaysia

**Keywords:** TiO_2_, green synthesis, *Deinbollia pinnata*, solvents polarities, methyl orange, photodegradation

## Abstract

Nanotechnology is one of the most interesting areas of research due to its flexibility to improve or form new products from nanoparticles (NPs), and as a fast, greener, more eco-friendly and sustainable solution to technological and environmental challenges. Among metal oxides of photocatalytic performance, the use of titania (TiO_2_) as photocatalyst is most popular due to its unique optical and electronic properties. Despite the wide utilization, the synthesis of TiO_2_ NPs bears many disadvantages: it utilizes various less environmental-friendly chemicals, high cost, requires high pressure and energy, and potentially hazardous physical and chemical methods. Hence, the development of green synthesis approach with eco-friendly natural products can be used to overcome these adverse effects. In this work, TiO_2_ NPs have been prepared by using *Deinbollia pinnata* leaves extracts, obtained by different solvents (*n*-hexane, ethyl acetate, and ethanol) with different polarities. The extracts acted as the reducing agent, while titanium isopropoxide as the precursor and water as the solvent. X-ray diffraction (XRD) pattern confirmed the synthesized TiO_2_ consist of anatase phase in high purity, with average crystallite size in the range of 19–21 nm. Characterization by using field emission scanning electron microscopy (FESEM) showed the TiO_2_ NPs possess a uniform semi-spherical shape in the size range of 33–48 nm. The energy dispersive X-ray (EDX) spectra of green TiO_2_ NPs showed two peaks for the main elements of Ti (61 Wt.%) and O (35 Wt.%). The band-gap energy of 3.2 eV was determined using UV-Vis spectroscopy. From the nitrogen sorption analysis, type V isotherm of the material was obtained, with BET surface area of 31.77 m^2^/g. The photocatalytic activity of synthesized TiO_2_ was evaluated for photodegradation of methyl orange (MO) under UV light irradiation. Based on the results, it is shown that TiO_2_ NPs synthesized with *D. pinnata* leaves extracted using ethyl acetate showed the most effective photodegradation performance, achieving 98.7% of MO conversion within 150 min. It can be concluded that the use of plant extracts in synthesis with TiO_2_ managed to produce highly crystalline anatase TiO_2_ with superior photocatalytic activity in the photodegradation of organic dye.

## Introduction

The nature of nanostructured materials has prompted natural product researchers' attention to this field. The naturally occurring oxide of titanium (IV) oxide or titania (TiO_2_) possesses unique optical and electronic properties (Ahmad et al., 2019). The surface chemistry of nanostructures in terms of smaller size and uniqueness have already been utilized in medicine, nutrition, energy (Venugopal et al., 2017; Agarwal et al., 2018; Kim et al., 2018) and in the biosynthesis of TiO_2_ NPs with metabolites present in plants extract to reduce and stabilize bulk metal into elemental forms (Mohamad et al., 2014). Plants are a major segment of biodiversity affected by many living and non-living components with their medicinal values. This is due to the presence of certain chemicals/active ingredients used for the treatment of disorders, with low cost, eco-friendliness and easy availability in the forest (Patel, 2015). Stable biological NPs are achieved with appropriate choice of solvents polarity for plant extraction (reducing agents) and as toxic-free material (Raghad et al., 2016). Fungi, actinomycetes, bacteria, and plant extracts (Órdenes-Aenishanslins et al., 2014) have replaced chemical agents in the green synthesis materials. Furthermore, the usage of these materials can enhance the photocatalytic and pharmacological properties of the NPs produced. Bio-mediated TiO_2_ NPs have also been obtained from the extracts of *Morinda citrifolia L*. (Sundrarajan et al., 2017), *Tamarindus indica* (Hiremath et al., 2018)*;* and *Azadirachta indica* (Thakur et al., 2019). The roots and leaves of *D. pinnata* are used in folkloric medicine as remedy for febrifuge, analgesic, bronchiasis intercostals, intestinal pains, jaundice, cough, asthma, and infections (Sotubo et al., 2016). Leaf extracts are used in fetus positioning during child birth (Kankara et al., 2015). The root act as an antibacterial agent (Sotubo et al., 2016).

Various synthesis methods of TiO_2_ NPs are available, such as solution combustion (Kitamura et al., 2007), sol-gel (Elbushra et al., 2018), hydrothermal (Bregadiolli et al., 2017), solvothermal (Dinh et al., 2012), microwave-assisted (May-Masnou et al., 2018), co-precipitation (Rab et al., 2018), and chemical vapor deposition (Lee et al., 2011). However, the sol-gel process clearly stood out due to the lower processing temperature (<100°C), which can produce highly crystalline particles with small sizes and high surface area. In addition, this method is also low in cost and can give fast composition homogeneity.

Dyes are major coloring agents used in industries for precious stones, paper, leather, plastic, textile, and food production. Hazardous waste water from dyeing process act as a major contaminant to the environment (Murugan and Parimelazhagan, 2014; Hejazi et al., 2020). The dye effluents are usually non-biodegradable, highly oxidizing, and stable to heat and light (Khataee and Mirzajani, 2010; Khataee et al., 2010) which attributed to their toxicity, undesirable aesthetic, and carcinogenicity (Khalik et al., 2017). This is due to their diversity and complex structure (Benkhaya et al., 2017), which need to be degraded. One example is methyl orange (MO), which is a commonly used dye.

Methyl orange dyes are stable under visible light, which can be easily degraded in the presence of catalyst (Chiu et al., 2019). It is known to cause irritation to reproductive, excretory, respiratory. and central nervous systems when in contact or consumed (Parida et al., 2013; Ljubas et al., 2015). Among all other methods to overcome its environmental hazard, photocatalysis has been proven effective for its degradation and removal of azo dyes in a less harmful (greener) method (Guettaï and Ait Amar, 2005). This present work focuses on the sol-gel green synthesis of TiO_2_ NPs using *D. pinnata* plant extracts obtained by different solvents with different polarities (*n*-hexane, ethyl acetate, and methanol). Their multi-functional group in the ethyl acetate crude extracts were characterized by Fourier transform infrared (FTIR) spectroscopy. The TiO_2_ NPs obtained were then characterized using X-ray diffraction (XRD), field emission scanning electron microscopy (FESEM), the energy dispersive X-ray (EDX), ultraviolet-visible light analysis (UV-Visible), nitrogen sorption analysis (N_2_ sorption), and photoluminescence analysis (PL). Following that, the photocatalytic activity of the synthesized TiO_2_ NPs was evaluated for the photocatalytic degradation of MO under UV light irradiation. This current work further shows the potential of utilizing plants extract in multi-fold directions, but such as in the case of biosynthesis.

## Experimental

### Materials

The materials used in this research were titanium isopropoxide (TTIP), C_12_H_28_O_4_Ti [Aldrich (97%)], methyl orange (MO) C_14_H_14_N_3_NaO_3_S [Aldrich (85%)], absolute ethanol (EtOH) C_2_H_6_O [MERK (100%)]. All materials were used as-received without further purification. Deionized water (MQ-H_2_O) was used throughout the experiments. Mettler H10, photoreactor chamber, membrane filter, syringe, centrifuge, UV lamp 1,200 W, glass beaker, cuvette, aluminum foil, and glass petri-dish were used as the apparatus in this work.

The *D. pinnata* (Poir.) Schumach. and Thonn leaves were collected from Okehi Local Government Area of Kogi State, Nigeria during dry season, early January, for 1 week (daytime temperature ranges from 28°C in January to 32°C). The plant was identified and confirmed at the Biological Department, Federal College of Education Okene Kogi State by Mrs. Aniama S.O.A., a botanist. The plant material was authenticated at Forestry Research Institute of Nigeria Ibadan through comparison with voucher specimen under accession number of FHI 3251. The leaves were collected, washed and air dried at room temperature for 1 month.

### Extraction

**Figure 1:**
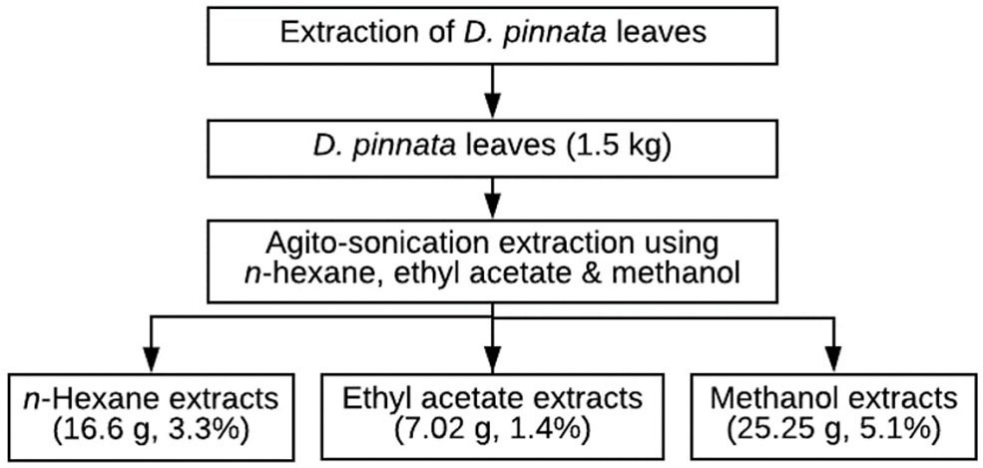
Extraction steps of *D. pinnata* leaves.

Powdered plant material of *D. pinnata* (Poir.) Schumach. and Thonn was extracted using sonication method as shown in Figure 1. Briefly, the leaf samples were placed into several conical flasks (30 g each) and extracted with organic solvents (150 mL), which were *n*-hexane, ethyl acetate, and methanol, in a sonicator using ultrasonic-assisted extraction method for 10 min with agitation. This method utilizes the following software conditions of time, temperature, and solvent ratio. Upon completion, the samples were filtered into bottles and allowed to settle for 24 h. The combined filtrates were then concentrated in *vacuo* at 40°C using a rotary evaporator.

### Qualitative Phytochemical Screening of Extract

The *D. pinnata* ethyl acetate extract was subjected to chemical tests for the identification of their active constituents. The chemical tests were performed using the methods reported by Samkeliso et al. (2018).

#### Test for Phenols

Five percent ferric chloride was added to 2 mL of the leaf extract, which will show black coloration. This indicates the presence of phenols.

#### Test for Flavonoids

Ten milliliters of ethyl acetate was added to the leaf extract and the test tube containing this mixture was heated in the water bath. One milliliter of diluted ammonia was added to this mixture, which will give yellow color, showing the presence of flavonoids.

#### Test for Alkaloids (Mayer's Test)

Mayer's test reagent was added to 2 mL of the leaf extract, which will then give cream colored precipitation, indicating the presence of alkaloids.

#### Test for Steroids

Two milliliters of concentrated H_2_SO_4_ was added to 1 mL of the extract. Following that, 2 mL of acetic anhydride was added. The change in the color of extract from violet to blue or green will take place if steroids are present.

#### Test for Coumarins

Ten percent of the NaOH was added to the extract and chloroform was added. Formation of yellow color will show the presence of coumarin.

### Synthesis of Green TiO_2_ NPs

The synthesis was carried out using the sol-gel method, where titanium (IV) isopropoxide (TTIP) was utilized as the precursor, *D. pinnata* extracts [1 g/5 mL] and deionized (DI) water as the medium. TiO_2_ NP_S_ were prepared by dissolving 4 mL TTIP with 20 mL of DI water under constant stirring for 30 min at 30°C. This was followed by the gradual addition of 0.2 g/mL of *DP* extracts (either by *n*-hexane, ethyl acetate, or ethanol) with constant stirring at 50°C for 4 h before kept at room condition for 48 h of aging process. The resulting colloidal suspension was washed with ethanol and centrifuged three times at 10,000 rpm for 30 min. The samples were then dried at 95°C for 24 h in an oven, before being calcined at 500°C in a muffle furnace for 5 h. The calcined TiO_2_ NPs powder was further characterized spectroscopically.

### Characterizations of Green TiO_2_ NPs

The crystallinity of the synthesized TiO_2_ NPs were performed by Rigaku Smart Lab, Germany with Cu Kα radiation (λ = 1.5418 Å) operated at 40 kV and 30 mA. The diffraction pattern was scanned in the 2θ range of 20–90°. The morphological features and surface characteristics of the photocatalyst were characterized using field emission scanning electron microscopy (FESEM) (JEOL JSM 6710F) equipped with EDX, at an accelerating voltage of 0.5–30 kV to determine the surface chemical element contents of the photocatalysts. The band gap energy of the TiO_2_ NPs were determined using UV-Vis near-IR (UV-Vis-NIR) spectrophometer (Shimadzu UV-3600 Plus UV-VIS-NIR). The spectra were recorded in the range of 300–500 nm at temperature rate/hold/time of 10:1,000:1.The specific surface area, micropore and pore size distribution of the TiO_2_ NPs were obtained using a multipoint Brunauer–Emmett–Teller (BET) analysis through the measurement of nitrogen adsorption–desorption isotherm as a function of relative pressure from a Quantachrome NOVA-4LX firmware with helium gas, and ambient temperature of 30.06°C. The thermal stability and heat flow were examined using thermogravimetric-differential scanning calorimetry analyzer (TGA-DSC, SHIMADZU DTG-60H). The scanning was performed within the furnace temperature range of 20–800°C with a heating rate and air flow rate of 10°C min^−1^ and 20 mL min^−1^, respectively. The electrons-holes recombination's of the photocatalysts through discrete electronic states were analyzed by Photoluminescence (Horiba-fluoroMax-4C spectrofluorometer) with emission of full spectrum range between 400 and 800 nm. The chemical functional groups were detected by Fourier transform infrared spectroscopy (1600 FT-IR). The spectra were recorded in the range of 400–4,000 cm^−1^ using PerkinElmer Spectrum One Spectrometer.

### Photocatalytic Testing

**Figure 2:**
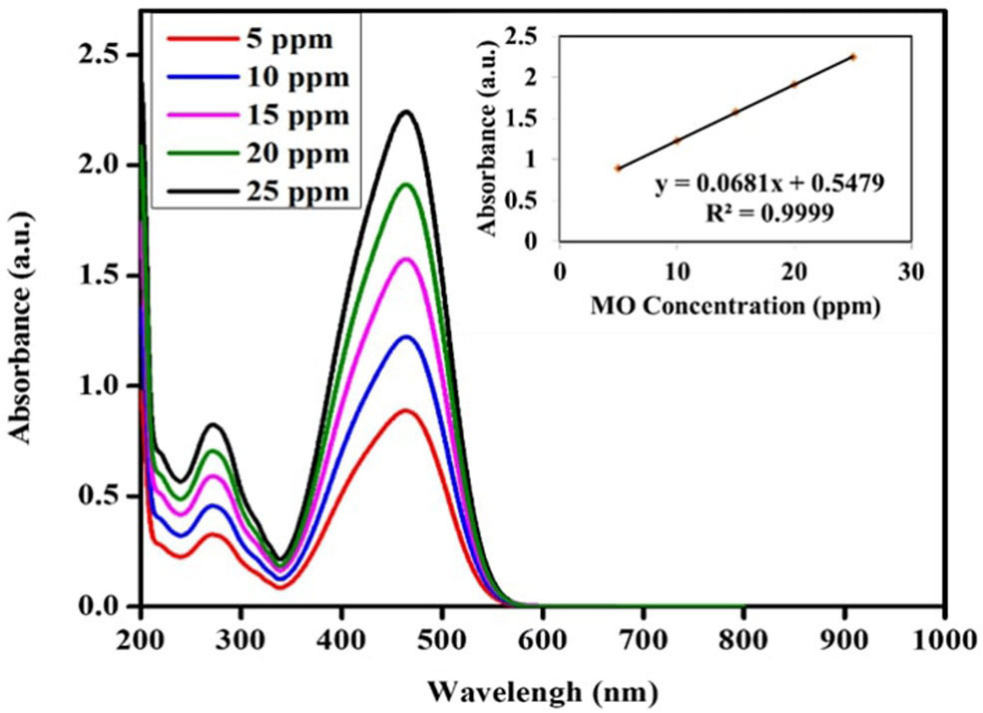
Calibration curve spectra and line graph of M.O. Solution.

The photocatalytic activity of the green TiO_2_ NPs was tested out in the degradation of methyl orange (MO, Aldrich 85%) using a 1,200 W lamp UV light source spaced at 3 cm horizontally. Prior to the test the stock solution of the water-based MO was prepared at 1,000 ppm in 100 mL and allowed to stand for 24 h. This was then followed by serial dilutions of 5, 10, 15, 20, 25, and 30 ppm. Five dilutions were used for the calibration curve, as shown in Figure 2, where *R*^2^ = 0.9999 was obtained from the straight line. For the reaction using the synthesized photocatalyst, the green TiO_2_ NPs synthesized was added to MO solution (50 mL) and sonicated. The solution was stirred using a magnetic stirrer in the dark condition for 1 h in order to reach the adsorption equilibrium. After reaching the adsorption equilibrium, the UV light was switched on 3 mL of the treated MO was collected after interval of 30, 60, 90, 120, and 150 min of reaction. The concentration of the MO from the withdrawn aliquot was analyzed using a UV-Vis spectrophotometer.

## Results and Discussion

### Possible Mechanism for the Formation of TiO_2_ NPs

Preliminary phytochemical screening was done using standard procedures to identify the constituents present in the extract, as described by Samkeliso et al. (2018). It involves observing plant samples for different classes of phytochemicals. This qualitative tests were used to provide an indication of the nature of phytochemicals present in D. *pinnata* (Poir.) Schumach. and Thonn's extract, as shown in Table 1.

**Table 1:** Phytochemical constituents present in ethyl acetate leaf extracts of *D. pinnata*.

**Constituents**	**Results**
Phenolics	+++
Flavonoids	+++
Alkaloids	++
Steroids	++
Coumarins	++

Table 1 shows that the plant of *D. pinnata* contains high amount of flavonoids and phenols that are important as surfactants and stabilizers. These constituents are able to prevent excessive agglomeration of the nanoparticulate material during the synthesis process. Other phyto-constituents present in the leaf extract were alkaloids, stereoids, and coumarins.

**Figure 3:**
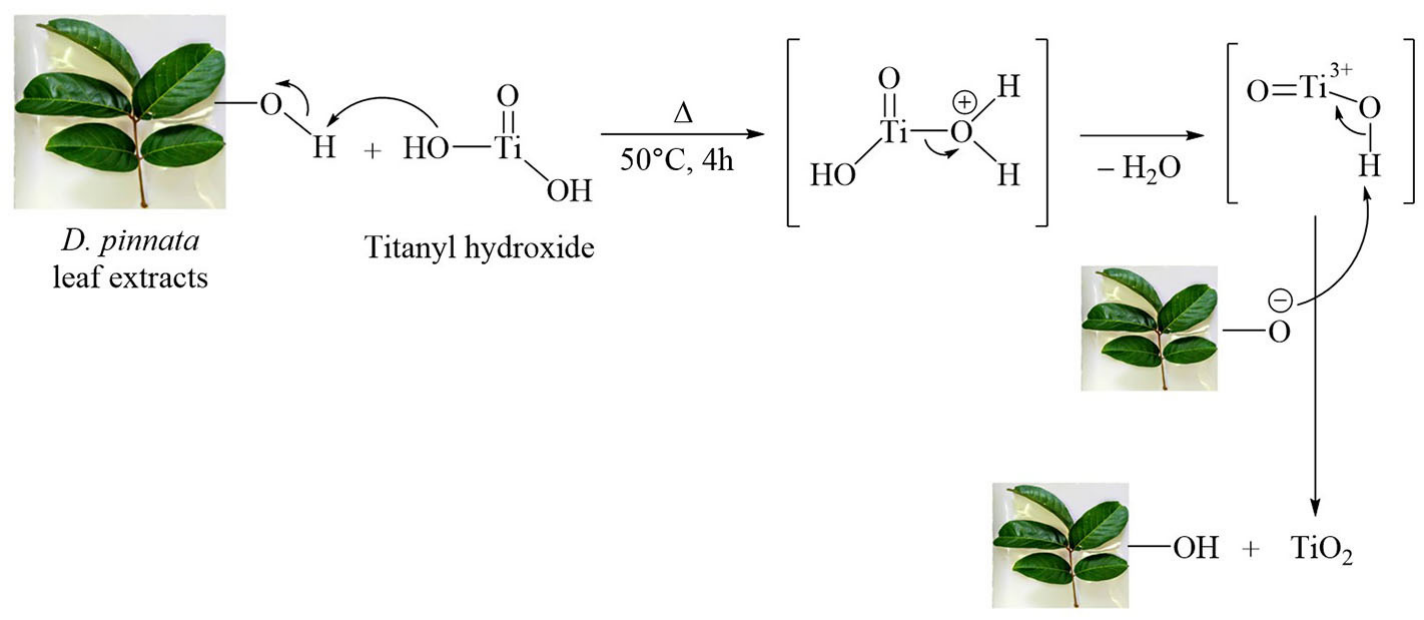
Possible reaction mechanism for the formation of TiO_2_ NPs in presence of hydroxyl group (–OH) of leaf extract of *D. pinnata*.

The mechanism for the formation of TiO_2_ is shown in Figure 3. TiO_2_ synthesis begins with complete hydrolysis of the precursor used [titanium isopropoxide, Ti(*i*-OPr)_4_] by direct interaction of the titanium source with water. Titanyl hydroxide was obtained according to the following reaction (Solano et al., 2019):

$$\begin{array}{l} Ti{\left(\text{i}-OC_{3}H_{7}\right)}_{4}+3H_{2}O\to TiO{\left(OH\right)}_{2}+\;4C_{3}H_{7}OH \end{array}$$

The FTIR data of *D. pinnata* ethyl acetate leaf extract displays the presence of a broad peak for OH functional group in the spectrum (Figure 13). It shows that the extract contains compounds having hydroxyl group as a functional group in the structure. Titanyl hydroxide can be dehydrated to give TiO_2_ nanoparticles by heating it with ethyl acetate leaf extracts of *D. pinnata* at about 50°C. In this process, *D. pinnata* extract serves as catalysts due to the present of compounds having hydroxyl group as a functional group in the structures (Selvaraj et al., 2012). Hence the reaction between *D. pinnata* extract and TiO(OH)_2_ might occur through the possible pathway which is described in Figure 3. Hence, water soluble compounds containing hydroxyl functional group are reported to be responsible for the stabilization of TiO_2_ nanoparticles.

### Physicochemical Properties Green TiO_2_ NPs

In this research, green TiO_2_ NPs were successfully prepared by the sol-gel method using *D. pinnata* plant, extracted by different solvents with different polarities (*n*-hexane, ethyl acetate, and methanol). First, the preparation of green TiO_2_ NPs colloid was obtained by gradual addition of extracts into the continuously stirred titanium isopropoxide in distilled water. Subsequently, TiO_2_ NPs were washed with absolute ethanol several times and calcined at 500°C, before being characterized.

**Figure 4:**
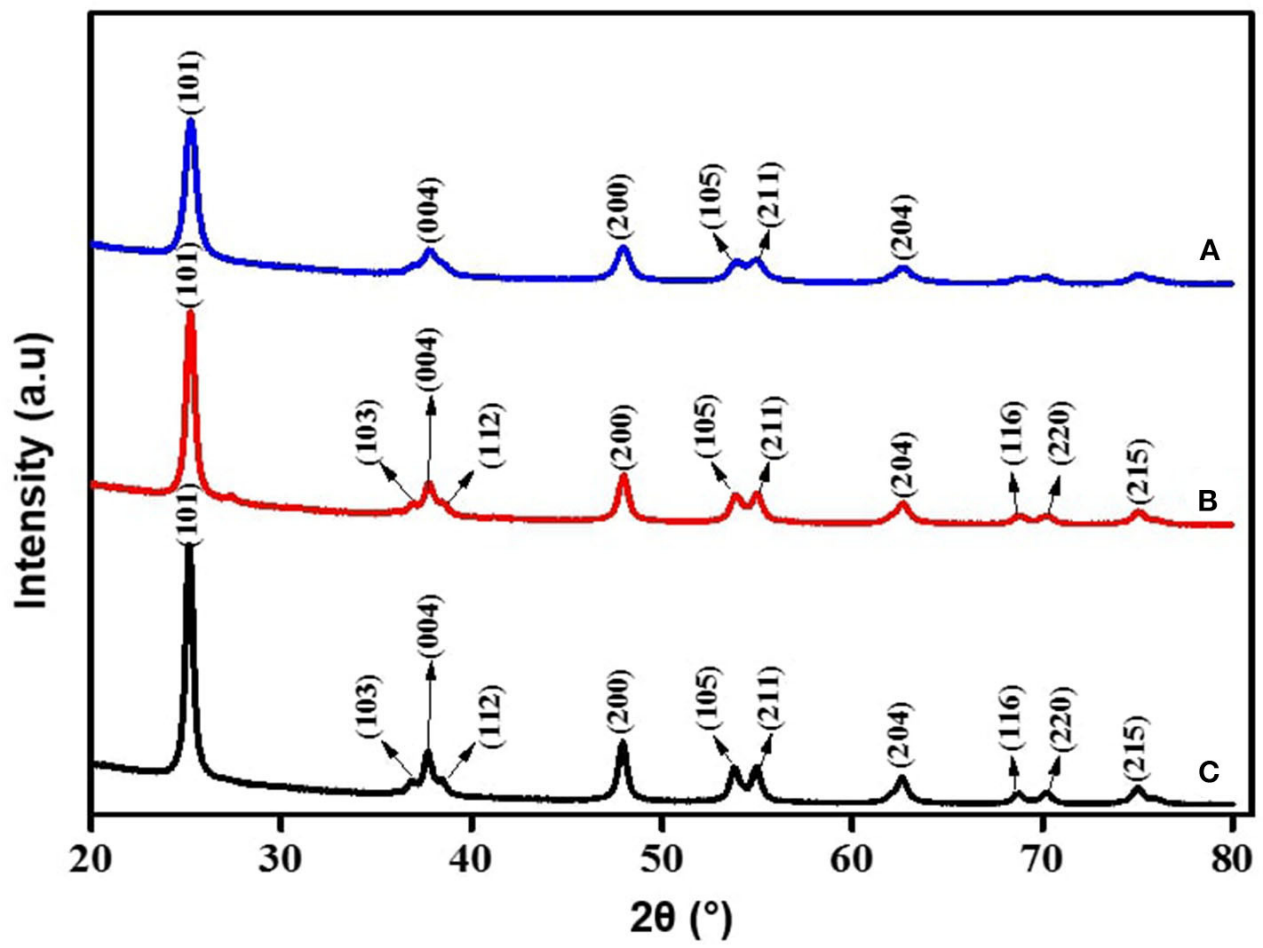
XRD patterns of green TiO_2_ NPs synthesized from the extracts of *D. pinnata* plant using **(A)** methanol, **(B)** *n*-hexane, and **(C)** ethyl acetate as the extraction solvents.

**Figure 5:**
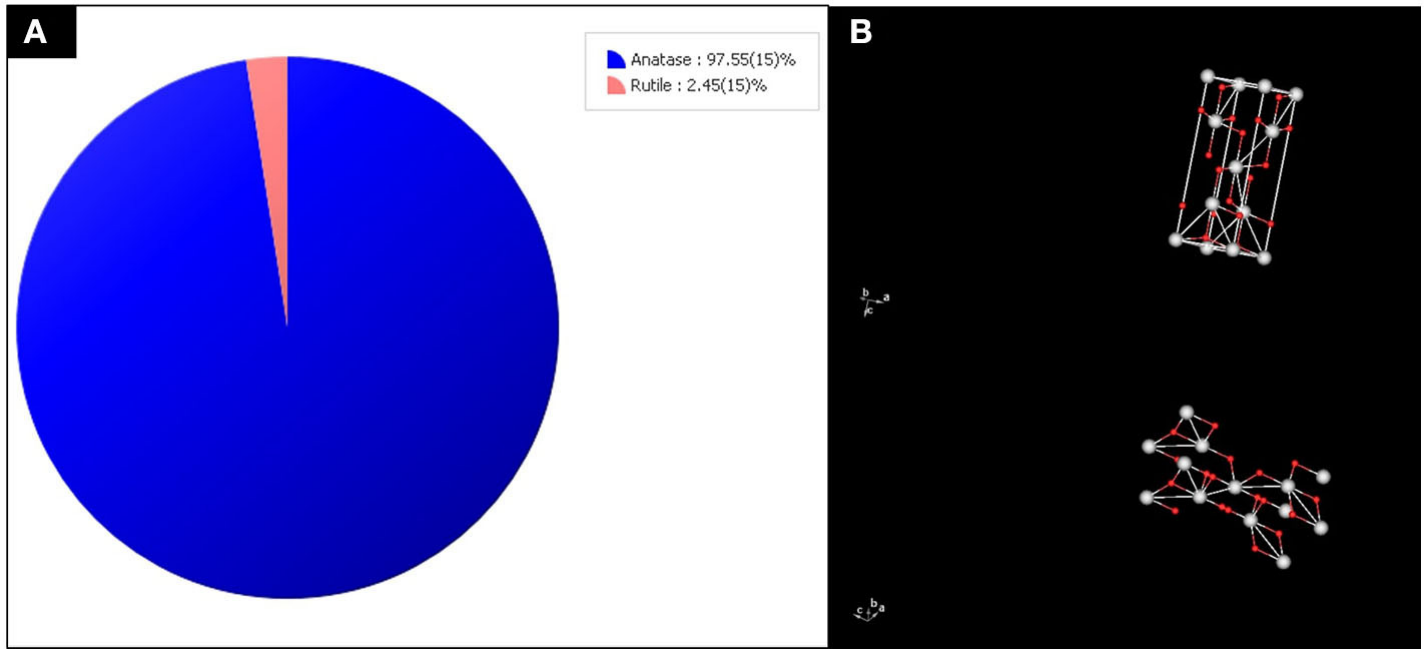
**(A)** The purity index and **(B)** 3D-tetragonal structure of anatase TiO_2_ obtained from the XRD results.

For the crystallinity studies done using XRD, as shown in Figure 4, the major peaks were at 2θ values of 25.18° (101), 37.73° (004), 47.94° (200), 53.76° (105), and 54.96° (211), which indicated the successful synthesis of anatase TiO_2_ NP_S._ The XRD pattern confirmed the synthesized TiO_2_ consist of anatase phase in high purity, with average crystallite size in the range of 19–21 nm. Scherer's equation (Lima et al., 2018), which relates the size of sub-micrometer crystallites in a solid to broadening of a sample peak in a diffraction pattern' illustrated as; *D* = *K*λ*/*β cos θ where (*D*) is average crystal size for the main broadening anatase peak; *K* = Scherrer's coefficient constant (0.89) which depend on the crystallite shape, λ = X-ray wavelength; β = full width at half maximum intensity (FWHM) of diffraction peaks in radians and θ = Bragg's angle, has been used to correlate the sample analysis reference pattern. The average crystallite size of the synthesized TiO_2_ was 11 nm, which showed similarity with TiO_2_ NPs synthesis using the extracts of *Syzygium cumini*, as reported by Sethy et al. (2020). The order of their lattice parameters is as follows: the fraction from ethyl acetate (a = 3.79632, b = 9.52847) > *n*-hexane (a = 3.79223, b = 9.51600) > methanol (a = 3.79237, b = 9.51330), which indicates the qualitative merit for the fraction obtained by ethyl acetate [(0.149) > *n*-hexane fraction (0.136) >methanol extracts (0.121)]. This is indirectly proportional to their 2θ vales of (101), where ethyl acetate fractions with 25.2324 < *n*-hexane fractions at 25.2607 < methanol fractions at the value of 25.2608. Thus, XRD morphological studies showed significant crystallinity, crystal phase, and peak sharpness of green TiO_2_ obtained using ethyl acetate plant extracts compared to those of methanol and *n*-hexane fractions, by comparing their major peaks, lattice parameter, and qualitative merit. Since TiO_2_ synthesized with the extracts of *D. pinnata* plant using ethyl acetate as solvent showed better results, further characterizations were focused on the ethyl acetate green TiO_2_ NP_S_. The crystallographic database (DB card number 5000223) did match with the tetragonal anatase of high purity indexed at 97.55 (15%), as displayed in Figure 5. The 3D-tetragonal crystal structure showed a supportive lattice parameter at a = 3.7963Å and c = 9.52847Å with angle of 90°.The quantitative analysis showed a figure of merit at 0.149 with a space group at 141: 141/amd:1. Such crystallinity, crystal phase, and peak sharpness of green TiO_2_ NP_S_ anatase were reported to influence their application as catalyst (Kim et al., 2018) or as support (Bagheri et al., 2014), acting as gas sensor (Zakrzewska and Radecka, 2017), degradation of pollutants (Phuinthiang and Kajitvichyanukul, 2019) and in solar cell (Kang et al., 2019).

**Figure 6:**
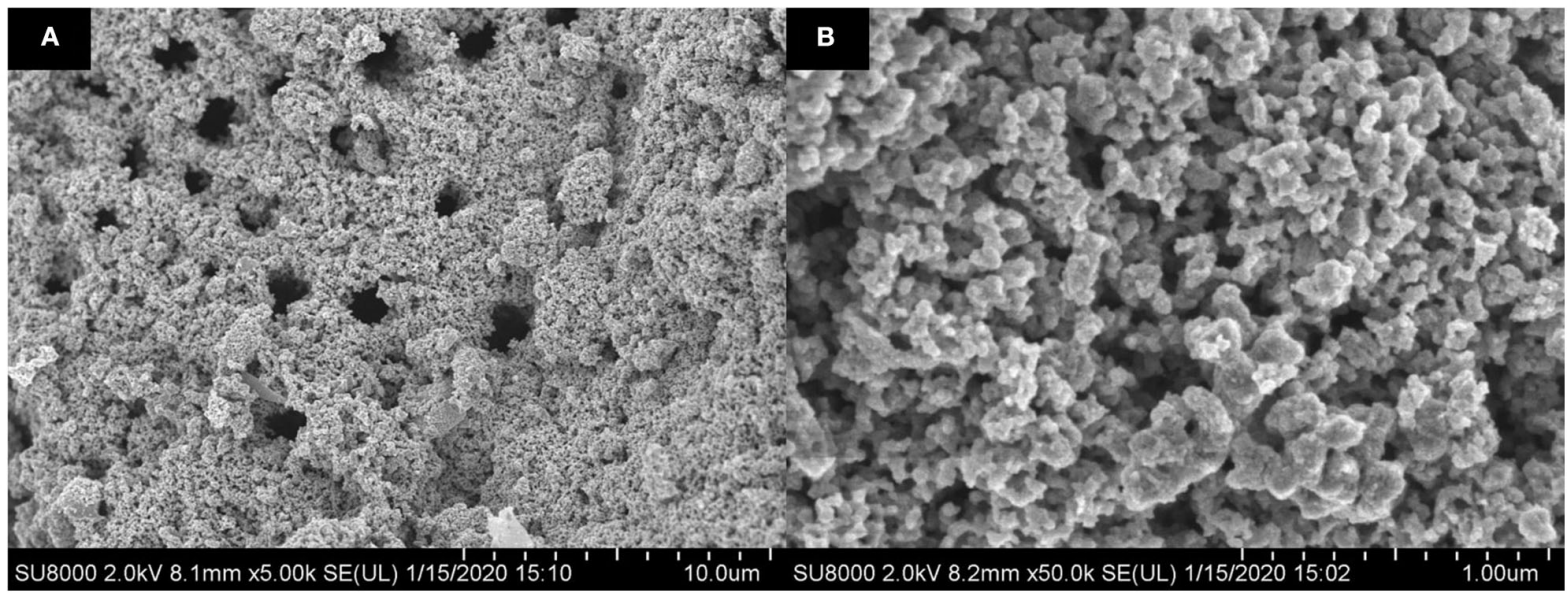
FESEM images of synthesized green TiO_2_ NPs with magnifications of **(A)** 5,000 times and **(B)** 50,000 times.

The FESEM images of green TiO_2_ NP_S_ synthesized from the extracts of *D. pinnata* plant, obtained by ethyl acetate, with different magnifications, are shown in Figure 6. The size of the particles were in the range of 19–21 nm, and the particles were densely aggregated, with non-uniformed semi-spherical shapes. This also proved that TiO_2_ particles in nano-sized have been successfully obtained.

**Figure 7:**
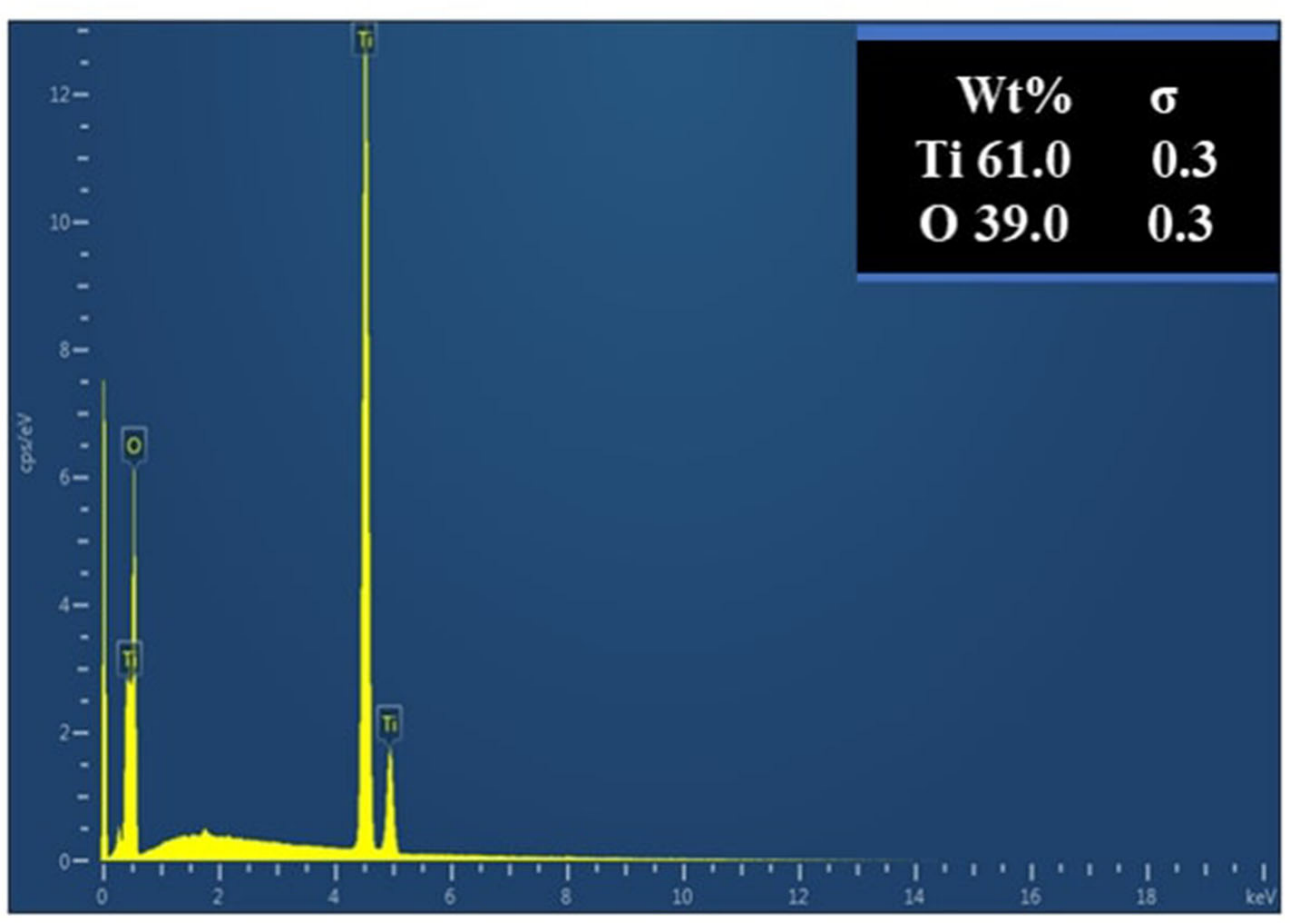
EDX spectrum of green TiO_2_ NPs synthesized from the extracts of *D. pinnata* plant using ethyl acetate as the extraction solvent.

The elemental analysis was performed via EDX. The EDX line scan spectrum of calcined TiO_2_ NPs in Figure 7 shows two peaks as the main chemical composition, which were Ti (61 wt.%) and O (35 wt.%). This is also consistent with the results obtained in the XRD analysis. The non-stoichiometric of TiO_2_ with the presence of oxygen can contribute to high performance in photocatalytic activity (Liu et al., 2012) with intense peak as bulk TiO_2_ and less intense peak as surface TiO_2_ (Ebrahimian et al., 2017). The EDX spectrum analysis showed that no other impurities were present in the synthesized green TiO_2_ NPs, which proved its high purity level.

**Figure 8:**
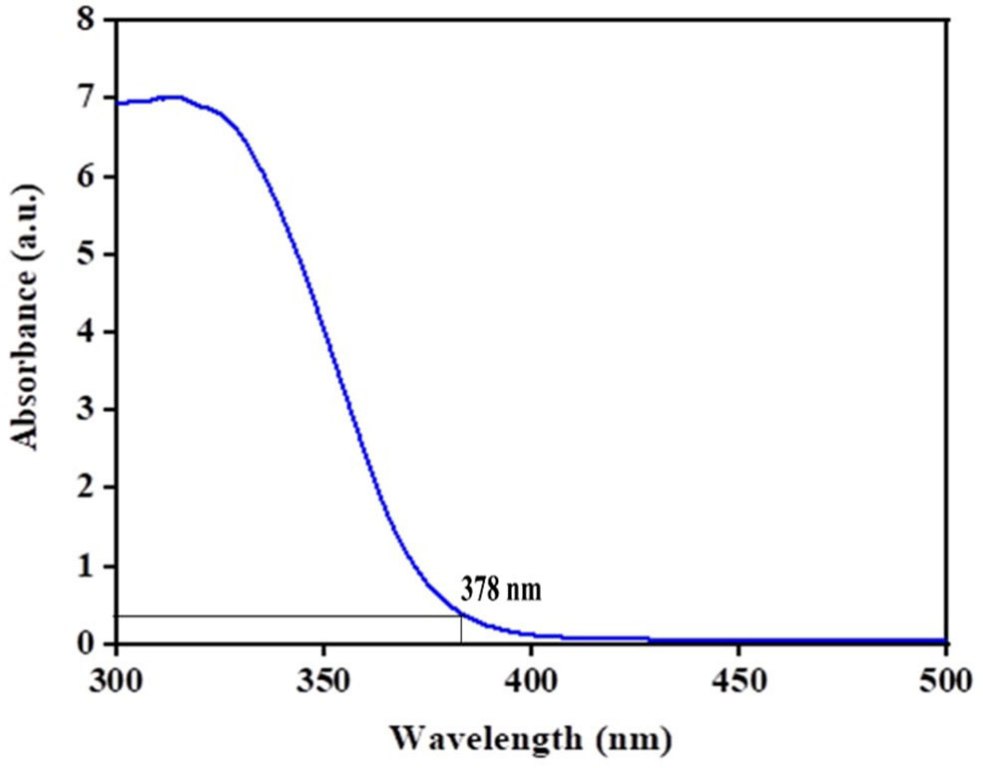
UV-Vis of green TiO_2_ NPs.

The optical absorption properties of the green TiO_2_ NPs were studied by UV-Vis-NIR as the absorption potential is an important factor to reflect its photocatalysis. The wavelength dependent absorbance was within the range of 300–500 nm. The band gap energy (*E*_*g*_) was calculated using this equation; *E*_*g*_ = 1239.8/λ in (Parida et al., 2013; Ljubas et al., 2015) where the absorption spectrum has a wavelength value of 378 nm, which indicated typical TiO_2_ anatase with high crystallinity as also reported by (Li et al., 2019), and band gap energy of 3.2 eV, as shown in Figure 8.

**Figure 9:**
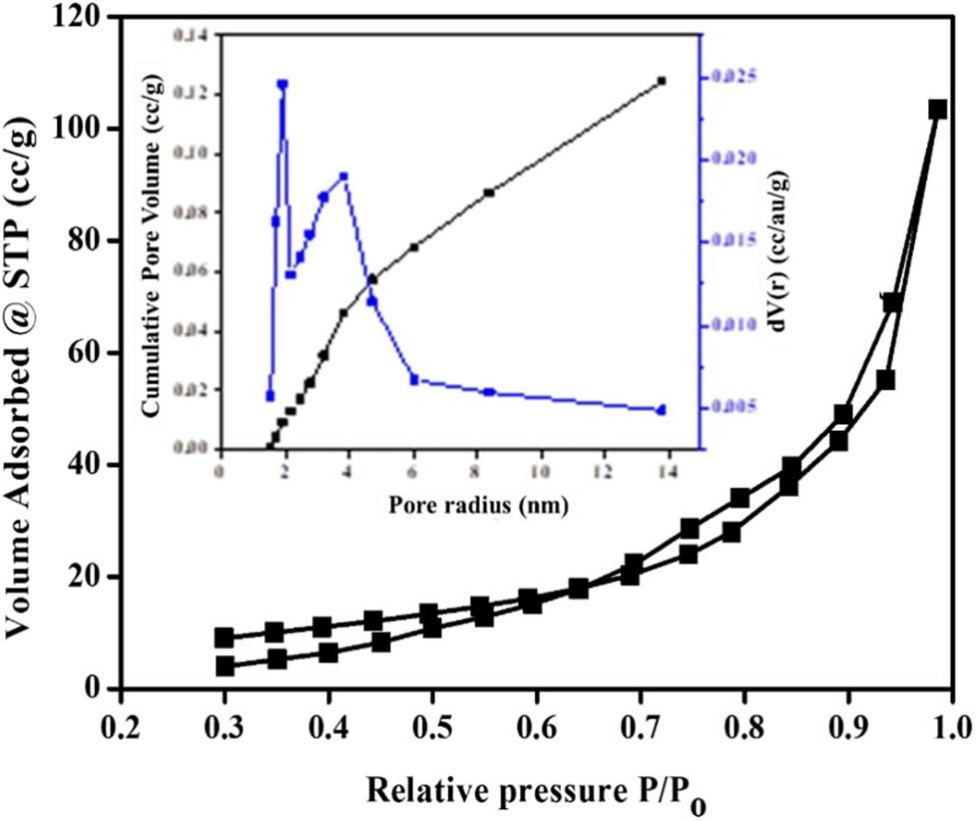
N_2_ isotherm and pore size distribution (inset) of the synthesized TiO_2_ NPs.

**Figure 10:**
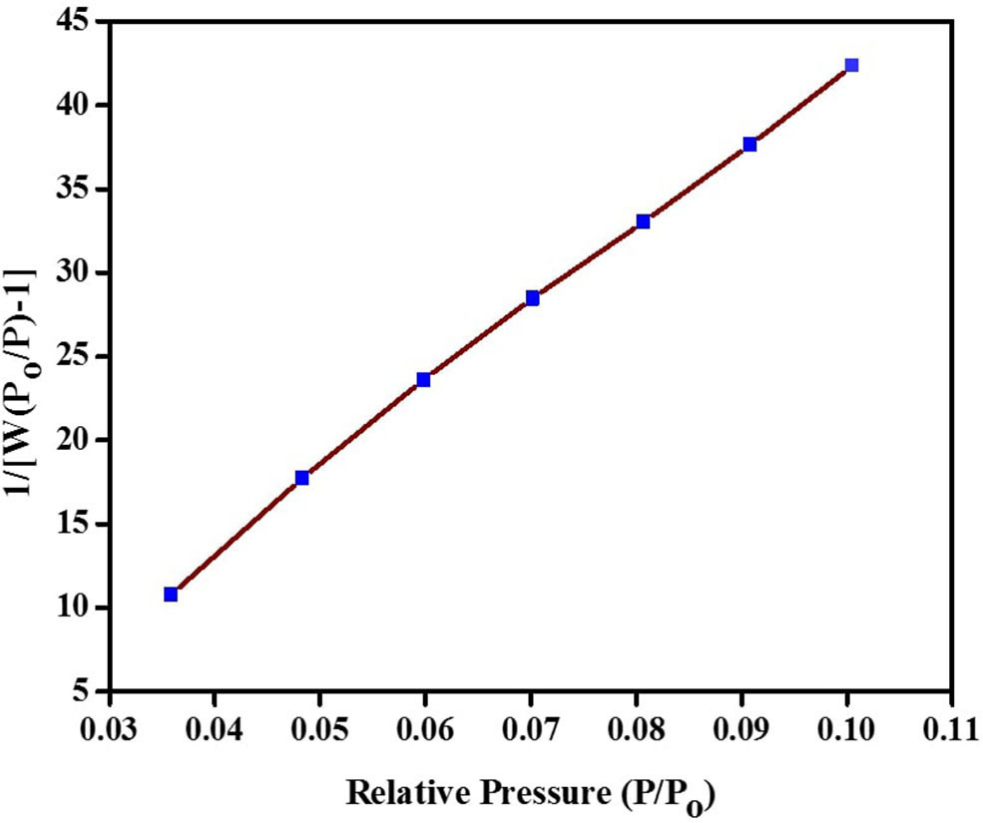
BET plot of the synthesized green TiO_2_ NPs.

The N_2_ isotherm and pore size distribution (inset) of the synthesized TiO_2_ NPs are shown in Figure 9. The isotherm is of type V isotherm, where a hysteresis loop can be seen. This indicates that the distribution of pores in an irregular flow rate (Tan et al., 2012). The BJH pore size distribution based on desorption branch showed major peaks starting from 2.02 to 6.00 nm with a broad peak at 3.98 nm, showing the mesoporosity. As for the specific surface area of synthesized TiO_2_ NPs, it was examined through BET multipoint graph, as shown in Figure 10. The BET surface area of the green TiO_2_ NPs was found to be 31.77 m^2^/g, which corresponds to correlation coefficient (*r*) of 0.99, as a liner graph (Ahmad et al., 2017).

**Figure 11:**
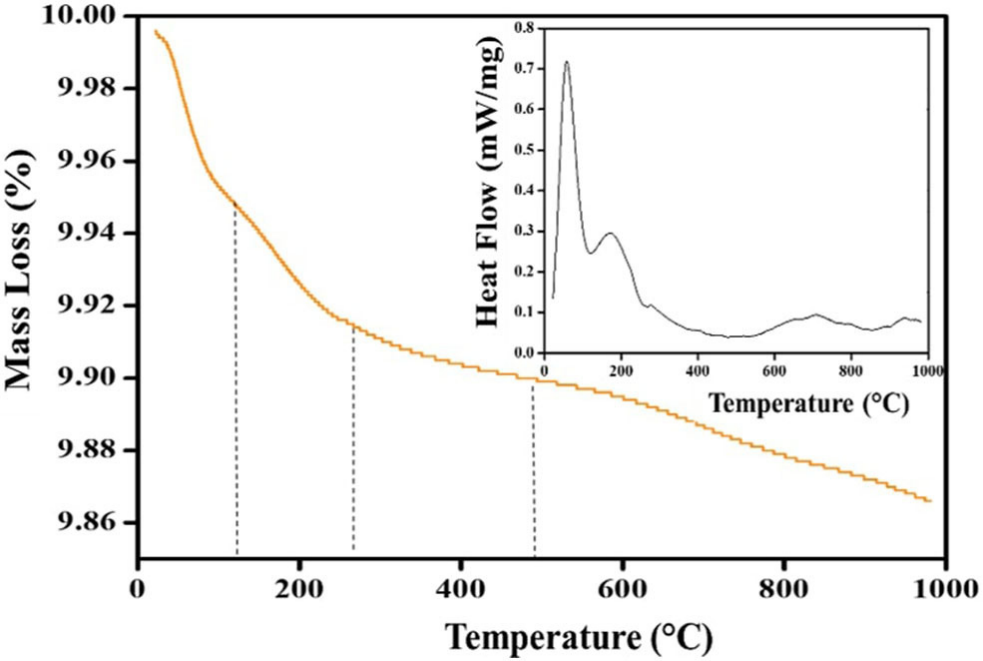
The TGA and DSC (inset) curves of the synthesized green TiO_2_ NPs.

Thermogravimetric analysis is a method used to determine thermal stability and the fractions of volatile components by monitoring the weight changes at a constant heating rate (Pintana and Tippayawong, 2013). The differential scanning calorimetry deals with the heat flow-temperature and this analysis has been carried out in the temperature range of 20–800°C using the uncalcined sample, as shown in Figure 11. The first weight loss band can be seen at 102°C, which is attributed to the loss of adsorbed atmospheric water (Yang et al., 2017). The second noticeable peak at 278°C was assigned to lower molecular weight metabolites, such as long chain fatty acids, galloyl ester or long chain esters. The last weight loss at 503°C marked the decomposition of all organic compounds (Kim et al., 2018). The irregular band line shown in the spectrum may be attributed to the nature of the products formed. The higher percentage mass loss (70%) was found for lower molecular weight constituents. The temperature at 106 and 288°C are associated with the loss of ethanol-water mixtures residue and fatty acids/ester endothermically. The exothermic peaks at 98 and 180°C seen on the DSC curve may be attributed to early phase transformation (Marinescu and Sofronia, 2011). The third region at 500°C justified total phase transformation of residual organic constituents of high molecular weight strongly coordinated to TiO_2_ atoms such as triterpenoids.

**Figure 12:**
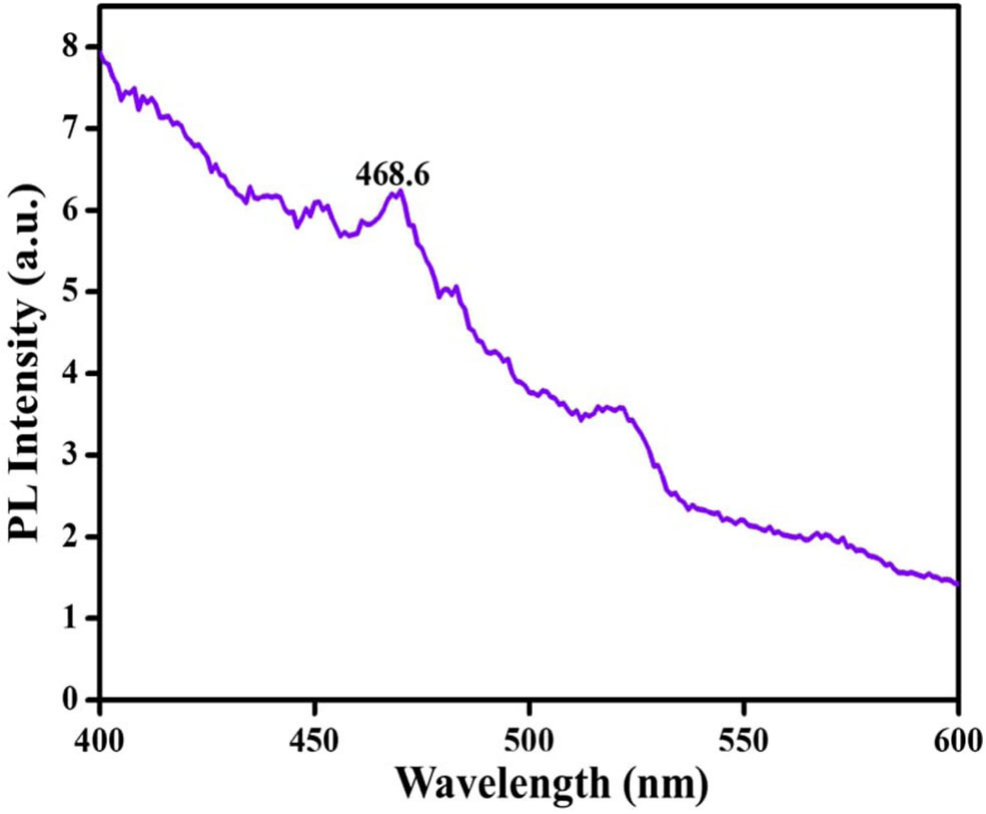
PL spectrum of the synthesized green TiO_2_ NPs.

Photoluminescence (PL) is also an important technique to study the optical properties of the synthesized TiO_2_ NPs. Figure 12 shows the p spectrum of the synthesized green TiO_2_ NPs. These NPs show a strong luminescent emission peak at 520.3 nm with irregular broad peak surfaces, in the green region, and a weak emission peak at 468.6 nm at room temperature. These transitions could be attributed to the complete inter-band formation in dxy orbital with phytoconstituents, implying 3d^2^ 4s^2^, and 3d^0^ in the Ti^4+^ ions (Yin et al., 2013). It was also observed that the second luminescence peak shifted toward the lower energy region. This may be attributed to the inter bonding of Ti-O from the carbonyl components in the plant signifying active anatase TiO_2_ of good light-response (Haghighatmamaghani et al., 2018). Furthermore, the shift toward the blue region may indicate a slight decrease in the particle sizes (Haque et al., 2017).

**Figure 13:**
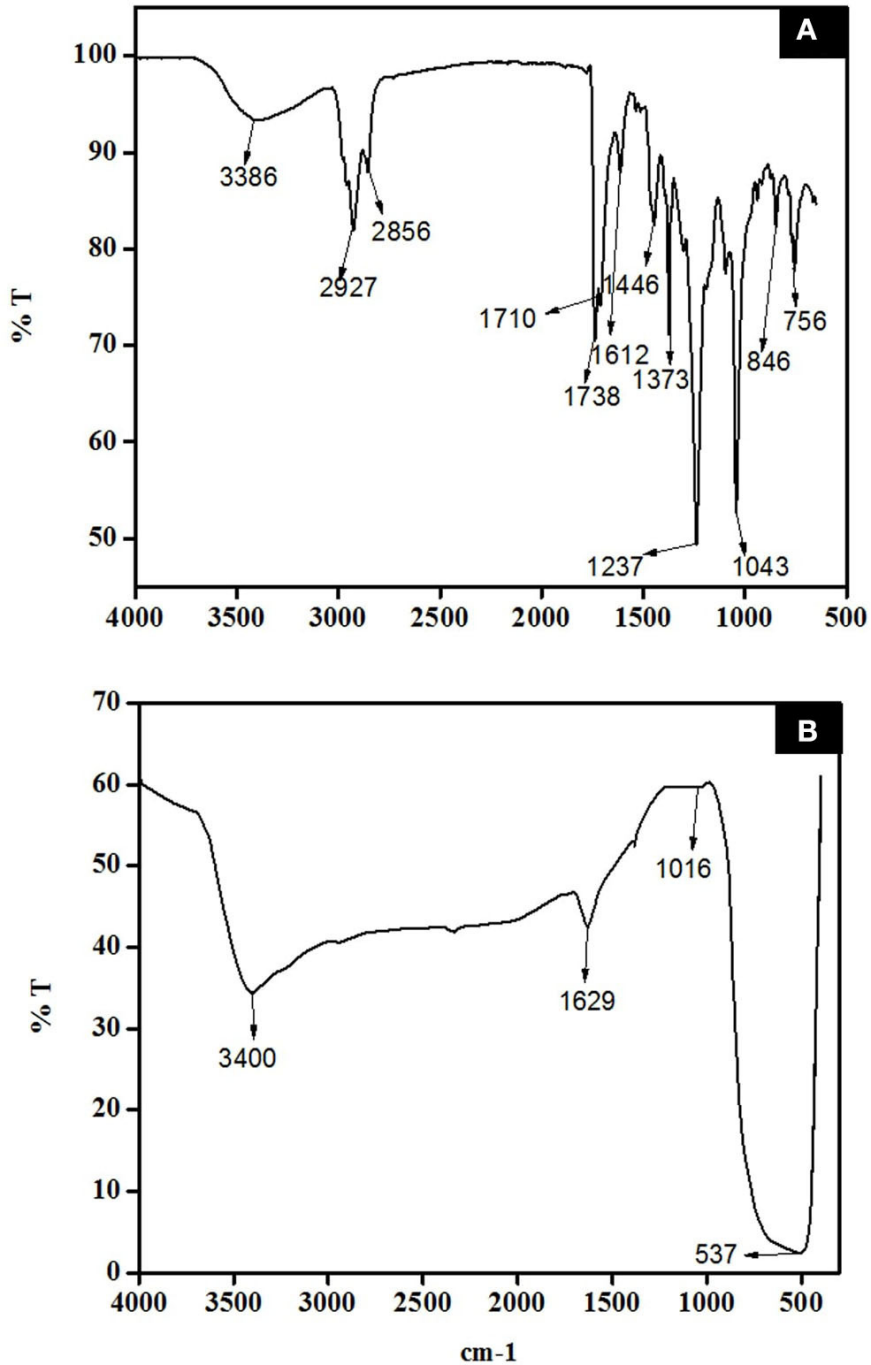
FTIR spectra of (**A**) *D. pinnata* crude extract and **(B)** synthesized TiO_2_ NPs.

The FTIR spectrum of *D. pinnata* ethyl acetate extract and TiO_2_ NPs were indicated by Figures 13A,B, respectively. Absorption bands at 3,386, 2,927, 1,710, 1,612, 1,446, 1,043, and 756 cm^−1^ are due to O-H bond stretching, C-H bond stretching, C=O bond stretching of carbonyl groups, C=C bond stretching, C-O bond bending vibration on phenolic compound, C-O bond stretching of the hydroxyl group, and out-of-plane C-H bending for aromatic ring. These functional groups represent the phytochemicals present in the extract. In Figure 12B, the broad absorption band observed at 3,386 cm^−1^ represents O-H bond stretching due to adsorbed moisture at the surface of TiO_2_ NPs. The broad band at 537 cm^−1^ represents a characteristic peak of Ti-O-Ti bending mode of vibration which confirms the formation of metal oxygen bonding (Catauro et al., 2018). The TiO_2_ NPs calcined at 500°C showed no traces of organic components functional groups, which justified complete calcination.

### Photocatalytic Activity

**Figure 14:**
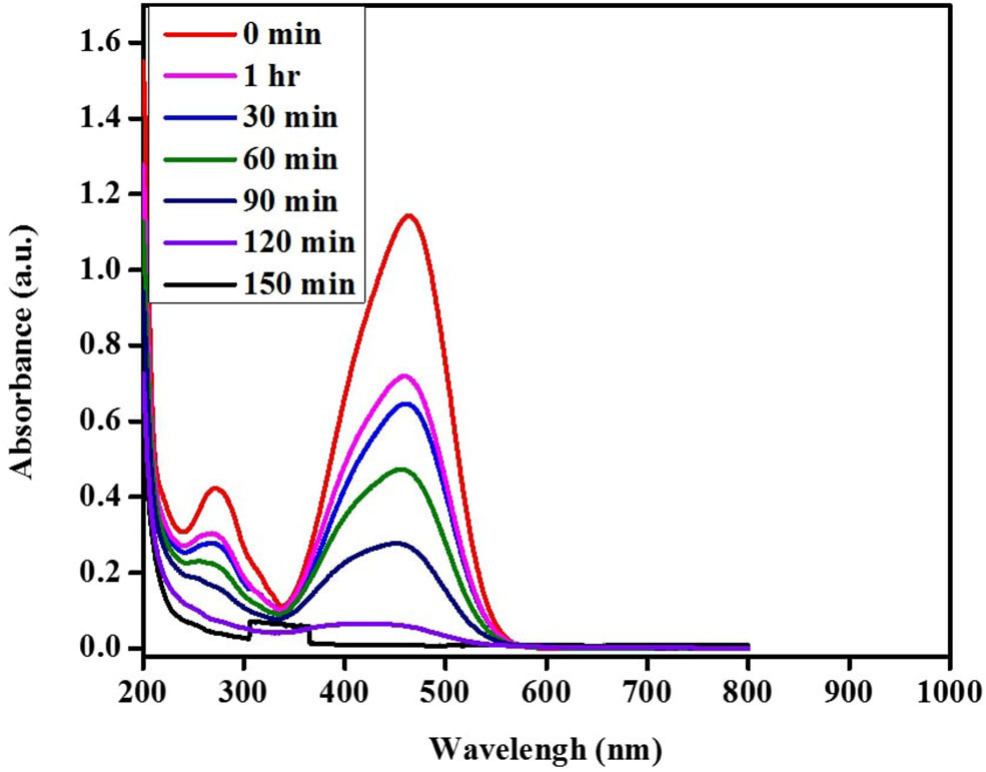
Absorbance spectra of photocatalytic degradation pattern of MO solution.

The photocatalytic activity of the green TiO_2_ NPs synthesized using the extracts of *D. pinnata* leaves with ethyl acetate as the solvent was tested out in the photodegradation of MO under UV light irradiation (1,200 W). The light source played a vital role in photocatalytic degradation as semiconductor compounds, such as TiO_2_, degrades volatile and organic pollutants under UV light irradiation (Cun et al., 2002; Laoufi and Bentahar, 2014; Luan et al., 2014). MO dye was chosen as the model organic pollutant and the photodegradation process was monitored by UV-visible spectrophotometer in the range of 800–200 nm. The degradation percentage was calculated using the following equation: *D* (%) = (*C*_*i*_-*C*_*f*_)/*C*_*i*_ × 100 where *C*_*i*_ is initial concentration, *C*_*f*_ is final concentration of MO at time (*t*) (Haque et al., 2017). Figure 14 shows the spectra of the reaction mixture withdrawn at intervals of 30, 60, 90, 120, and 150 min. After only 90 min, 51.72% of MO has been degraded. It was also observed that at 120 min, the photodegradation reached 81.36%, while at 150 min, 97.53% of MO photodegradation was obtained. It is evident that the rate of photodegradation of green TiO_2_ NPs photocatalyst on MO increased with longer irradiation time. This indicate faster interaction between the photons and green TiO_2_ NPs, which showed direct proportionality to the duration of irradiation. Therefore, MO (20 ppm) showed optimum irradiation duration of 150 min (97.53%). This can signify rapid formation of ∙OH radicals as active sites on the TiO_2_ NPs' surface for effective adsorption throughout the reaction (Youssef et al., 2016). Based on these results, it can be seen that an optimal performance of green TiO_2_ NPs can be achieved within a short time. This may indicate that extracts from plants as green substitute for the synthesis of NPs has the benefits of enhancing the charge carrier's separation (Solano et al., 2019), increasing specific surface area and overall photocatalytic performance. The physicochemical properties and photocatalytic activity have justified the efficient performance of green synthesized TiO_2_ NPs from *D. pinnata* plant for the photodegradation of MO.

## Conclusion

Based on the physicochemical characterization, TiO_2_ NPs have been successfully synthesized using the extracts of *D. pinnata* leaves. Different solvents used in the extraction also showed the contributions of solvents polarity in the extractions, where in this work, extracts using ethyl acetate showed superior characteristics. The synthesized green TiO_2_ NPs also showed good photocatalytic activity in the photodegradation of methyl orange dye under UV light irradiation. The work here showed that TiO_2_ NPs with good physicochemical properties and photocatalytic activity can be synthesized using extracts of plants, which can reduce or eliminate the use of harmful chemicals. Further work on the physicochemical properties and photocatalytic activity of TiO_2_ NPs obtained using extracts from *n*-hexane and methanol will be carried out.

## Data Availability Statement

The original contributions presented in the study are included in the article/supplementary materials, further inquiries can be directed to the corresponding author/s.

## Author Contributions

All authors listed have made a substantial, direct and intellectual contribution to the work, and approved it for publication.

## Conflict of Interest

The authors declare that the research was conducted in the absence of any commercial or financial relationships that could be construed as a potential conflict of interest.
